# Immunotherapy and Epigenetic Pathway Modulation in Glioblastoma Multiforme

**DOI:** 10.3389/fonc.2018.00521

**Published:** 2018-11-13

**Authors:** Christopher Chin, Emma S. Lunking, Macarena de la Fuente, Nagi G. Ayad

**Affiliations:** ^1^Department of Psychiatry and Behavioral Sciences, Center for Therapeutic Innovation, Sylvester Comprehensive Cancer Center, Miami Project to Cure Paralysis, University of Miami Miller School of Medicine, Miami, FL, United States; ^2^Department of Neurology, University of Miami, Miami, FL, United States; ^3^Sylvester Comprehensive Cancer Center, University of Miami, Miami, FL, United States

**Keywords:** glioblastoma, immunotherapeutic, clinical trial, epigenetic, long non coding RNA

## Abstract

Glioblastoma Multiforme (GBM) is the most common malignant primary brain tumor. Despite aggressive multimodality treatment it remains one of the most challenging and intractable cancers (1]. While current standard of care treatment for GBM is maximal safe surgical resection, systemic chemotherapy with Temozolimide (TMZ), and radiation therapy, the current prognosis of GBM patients remains poor, with a median overall survival of 12–15 months (2, 3). Therefore, other treatments are needed to provide better outcomes for GBM patients. Immunotherapy is one of the most promising new cancer treatment approaches. Immunotherapy drugs have obtained regulatory approval in a variety of cancers including melanoma (4), Hodgkin lymphoma (5), and non-small cell lung cancer (6). The basis of immunotherapy in cancer treatment is linked to stimulating the immune system to recognize cancer cells as foreign, thereby leading to the eventual elimination of the tumor. One form of immunotherapy utilizes vaccines that target tumor antigens (7), while other approaches utilize T-cells in patients to stimulate them to attack tumor cells (8). Despite intensive efforts all approaches have not been overtly successful (9), suggesting that we need to better understand the underlying biology of tumor cells and their environment as they respond to immunotherapy. Recent studies have elucidated epigenetic pathway regulation of GBM tumor expansion (10), suggesting that combined epigenetic pathway inhibition with immunotherapy may be feasible. In this review, we discuss current GBM clinical trials and how immune system interactions with epigenetic pathways and signaling nodes can be delineated to uncover potential combination therapies for this incurable disease.

## Review of clinical trials using combination immunotherapy

Multiple immunotherapy clinical trials in GBM have been initiated although few have reached completion. And of those few, the results observed were limited. For example, an upfront (newly diagnosed GBM) phase 2 trial tested the efficacy of a patient specific dendritic cell vaccine termed ICT-107, by observing its ability to significantly change the median survival rate of newly diagnosed GBM patients. In the ICT-107 study, patients in the experimental arm as well as control arm underwent surgical resection followed by radiation and TMZ treatment for six weeks. Subsequently, white blood cells (WBCs) from GBM patients were extracted from both experimental and control groups and cultured with antigens found in the GBM experimental group and not in the control group. Over the period of several months, the WBCs pulsed with GBM antigen for the experimental arm and WBCs not pulsed with GBM antigen for the control arm were reintroduced as vaccines to patients in the respective groups. The results showed that when compared to patients in the control group, whose extracted WBC were not cultured with GBM antigens, the GBM cultured WBCs increased survival in ICT-107 treated patients by only less than 2 months [Table 1, (23)].

**Table 1:** A summary of clinical trials utilizing standard of care for GBM in addition to immunotherapy and biomarker immunotherapy in combination with and without targeted therapy.

	**Immunotherapy**	**Biomarker immunotherapy**
Standard of care, targeted therapy, and immunotherapy: immunotherapy combined with other treatment method(s)	Combination of immunization and radiotherapy for recurrent GBM (InSituVac1) (InSituVac1) (11) Basiliximab in treating patients with newly diagnosed glioblastoma multiforme undergoing targeted immunotherapy and temozolomide-caused lymphopenia (REGULATe) (12) Vaccine therapy in treating patients with newly diagnosed glioblastoma multiforme (ACTIVATe) (13) Phase II feasibility study of dendritic cell vaccination for newly diagnosed glioblasto mamultiforme (14) A study of ICT-107 immunotherapy in glioblastoma multiforme (GBM) (15)	Tremelimumab and durvalumab in combination or alone in treating patients with recurrent malignant glioma (16) Pembrolizumab and vorinostat combined with temozolomide for newly diagnosed glioblastoma (17) Adjuvant dendritic cell immunotherapy plus temozolomide in glioblastoma patients (ADDIT-GLIO) (18)
Immunotherapy and standard of care:	Immunotherapy for patients with brain stem glioma and glioblastoma (19) Tumor lysate pulsed dendritic cell immunotherapy for patients with brain tumors (20) Dendritic cell-based tumor Vaccine adjuvant immunotherapy of human glioblastoma multiforme (WHO grade IV gliomas) (21) A pilot study to evaluate PBR PET in brain tumor patients treated with chemoradiation or immunotherapy (22)	A study of ICT-121 dendritic cell vaccine in recurrent glioblastoma (19)

Other combination trials using immunotherapy with chemotherapy and/or radiotherapy have been performed in the hopes of attaining statistically significant results, but most have fallen short. Another upfront phase 2 immunotherapy combination trial investigated the effect of immunotherapy with radiation and chemotherapy to determine the efficacy of combination therapy as treatment for GBM with the PEP-3-KLH vaccine for newly diagnosed EGFRvIII-expressing GBM patients. The PEP-3-KLH vaccine is a synthetic peptide derived from a mutated segment of the epidermal growth factor type vIII (EGFRvIII), which is overexpressed in some patients with malignant glioma (24). This mutated segment is then conjugated onto the adjuvant keyhole limpet hemocyanin (KLH), a respiratory protein that is similar to some GBM antigens. The researchers in this study used this vaccine to assess its synergistic ability to elicit an immune response in conjunction with radio- and chemo-therapies when compared to patients who only received radio- and chemotherapies alone. In the first of three arms of this study, the PEP-3-KLH vaccine was administered to the GBM patients after they had completed radiation treatment. In the second and third arms of this study, the PEP-3 vaccine was used after radiation in combination with the oral chemotherapy drug, TMZ (25). The patients in this study who received TMZ, radiotherapy, and vaccine were compared to a matched cohort who were only treated with radiation therapy and TMZ. When compared to the median overall survival (OS) of the matched cohort, which was 15 months (95% CI 11.4 to 19.7 months), the vaccinated patients had an increased median OS of 26 months (95% CI, 21.0 to 47.7 months) (26). While these results are promising, it should be noted that only a subset of GBM patients have the EGFRvIII antigen on their GBM tumor cells, which was present in all participants studied (26). In fact, a study discussing the prognostic significance of EGFRvIII antigen found that only 14 of 73 (19.2%) patients evaluated with primary GBM expressed the EGFRvIII antigen (27). Collectively, these findings suggest that only a select few GBM patients have the potential to benefit from this treatment.

**Figure 1:**
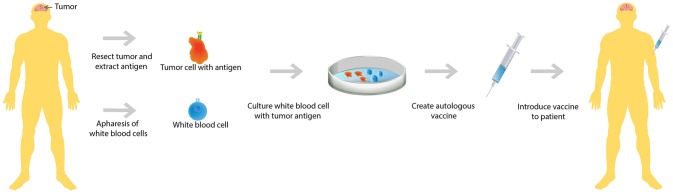
Autologous vaccine therapy. Both GBM tumor antigen and white blood cells are extracted from the patient. Subsequently, the extracted white blood cells are cultured with the GBM tumor antigens. A vaccine is created that is specific to each individual GBM patient's tumor antigen and then the cultured white blood cells are reintroduced to the patient.

A second upfront combination trial analyzed the efficacy of immunotherapy in conjunction with radiotherapy and chemotherapy. In this trial, patients who had recently undergone tumor resection for their newly diagnosed GBM were first treated with radiotherapy and concurrent chemotherapy, TMZ. After completion of both radiotherapy and chemotherapy, WBCs were collected from these patients and cultured along with dendritic cells with each individual patient's GBM tumor antigens (Figure 1). This autologous dendritic cell vaccine was reintroduced to each patient whose GBM antigens were used to culture the vaccine (28). This study showed that only 50% of this carefully selected patient population mounted an immune response resulting in improved survival. In addition, the study noted that there was vast heterogeneity in the immune response (29), meaning that the immune reaction to treatment is different in each individual.

The latter two upfront GBM trials mentioned above suggest a common theme in the ability to properly treat GBM. Patient outcome is highly variable, which could possibly be due to the heterogeneity of GBM tumors. In 2010, research utilizing data from The Cancer Genome Atlas developed a catalog of genomic abnormalities correlated with GBM tumors and helped to categorize four different GBM tumor subtypes: Classical, Proneural, Mesenchymal, and Neural (30), which has been more recently reclassified into Classical, Proneural, and Mesenchymal as the 3 main the subtypes of GBM (31). The significance of GBM subtype lies within the discovery that each subtype holds distinct genomic abnormalities, tumor microenvironments, and most importantly in terms of patient outcome, treatment response. Furthermore, GBM subtype switching has been observed upon disease recurrence, such as a Proneural to Mesenchymal transition, which been implicated in treatment resistance GBM (32). Thus, GBM subclasses have the potential to influence patient treatment, though the clinical relevance of this proposed classification remains to be determined. This is especially true given recent single cell sequencing of GBM tumors demonstrating that the even within classes of tumors vast heterogeneity exists (33–35). Future single cell sequencing studies will delineate the cell populations that remain after immunotherapy in order to better design combination therapies.

In comparison to upfront GBM trials for combination immunotherapy, recurrent GBM trials have even fewer results. These trials include single therapy dendritic cell vaccines in which patient WBCs are cultured and reintroduced (similar to the ICT-107 trial mentioned above) (19), treatment with immune adjuvants with radiation (11), and combination treatments utilizing checkpoint inhibitors (discussed below) (16). Overall, while there are some other recurrent GBM clinical trials, few have reported results.

## PD-L1 immune system blockade and checkpoint inhibition

The programmed death pathway's role in GBM tissue immunity has been examined and preclinical studies suggest that inhibition of this pathway, termed “checkpoint inhibition,” has high therapeutic potential. This pathway involves down regulation of the immune response after it has run its course (36). Specifically, in a healthy individual, it stimulates the inhibition of function in T-cells, and thus prevents T-cells from being overactive in an individual whose immune response needs to be stopped after an infection (37). In the case of tumors, activation of this pathway prevents T-cells, which express the PD-1 receptor, from accessing and acting on tumors. In mice transplanted with human GBM tumors, anti-PD-1 therapy completely eradicated GBM in 44.4% of mice and combination anti-PD-1 therapy with the chemotherapy drug TMZ completely eradicated GBM tissue in all mice. Combined therapy also reduces the frequency of exhausted tumor infiltrating lymphocytes (TIL), or T-cells that have lost their functional ability to develop immune responses (38), without affecting the non-exhausted TIL load that penetrate the BBB and enter GBM tissue. In other words, after combined therapy TIL (CD8^+^ T-cells) do not highly express the PD-1 receptor and therefore, cannot bind the PD-L1 (ligand) on GBM tissue resulting in the high success rate of this combined therapy (39). However, while these results boast an extremely potent immunotherapy regimen in mice, they have not translated well into efficacy in GBM clinical trials.

One issue that needs to be addressed in immunotherapy trials is the immunosuppressive microenvironment of GBM (40), which can induce angiogenesis leading to tumor growth (41). The PD-L1 ligand can induce and upregulate T-reg cells (42), which are immunosuppressive cells that protect the GBM tumor from the body's immune system (43). T-reg cells are involved in the inhibition of T-cells that recognize and attack self-antigen that are present on both normal tissue and tumor tissue (44). In a normal setting, this prevents a host's immune system from developing autoimmunity, or the condition in which the immune system attacks its own cells. In the case of GBM, induction of T-reg cells prevents the destruction of the tumor tissue due to presence of self-antigen on tumor cells.

The first large scale anti-PD-L1 therapy clinical trial in GBM was conducted using a drug called Nivolumab. Unfortunately, unlike the mouse studies mentioned above, the results were not promising. Specifically, nivolumab was determined to be no more effective at increasing overall survival than the anti-VEGF cancer drug, bevacizumab (37). But this lack of success was not a failure because it highlighted a few key points about the immunosuppression of GBM. It appears anti-PD-L1 therapy may not be sufficient to alleviate immunosuppression of GBM patients. In other words, other immunosuppressive factors within the microenvironment of GBM may render T-cells anergic, or unable to produce a functional response, which would result in a failed immune response despite PD-1 pathway inhibition. In addition, this highlights the blood brain barrier (BBB) as a significant limiting factor in GBM treatment. Because nivolumab is too large to cross the BBB, this study supports the assumption that anti-PD-L1 antibody therapy exerts its effect outside of the BBB. The therapeutic effect of anti-PD-1 treatment on T-cells occurs before they cross the BBB and enter the tumor microenvironment. However, if there is an inadequate population of T-cells in the periphery and/or the T-cells have already crossed the BBB and have been rendered anergic by the microenvironment of the GBM tumor, then PD-L1 therapy may not be effective, as was exemplified in this trial (37).

## Epigenetics

As discussed above, immunotherapy and combined immunotherapy are intensively studied in the treatment of GBM, although they do not seem to be wholly effective in treating GBM nor do they encompass the entire picture of GBM treatment. Recent discoveries have identified epigenetic pathway specific GBM biomarkers, which have enormous potential in terms of treatment because they can be used as targets for therapy. This epigenetic focus encompasses genes and gene regulators that are not dependent on the DNA sequence yet can be inherited and modified by endogenous enzymes. The influence of these epigenetic enzymes facilitates DNA modifications like methylation, acetylation, phosphorylation, and ubiquitination, all of which alter gene expression and change the state of cells within the body. Ultimately, dysfunction in these enzymes can lead to modifications within the cell that can lead to the development of cancer (45).

However, there is hope regarding epigenetic modifications that result in cancer. The good news is that modification of the DNA sequence by epigenetic enzymes is reversible. In fact, it appears as though a key factor in epigenetic regulation are long noncoding RNA transcripts (lncRNAs) that facilitate the molecular processes of epigenetic regulation (46). lncRNAs have been proposed to control activation and modulation of epigenetic enzymes (46, 47), and lncRNAs have been shown to be involved in cancer resistance to immune reaction through antigen release, antigen presentation, immune activation, and immune cell migration and infiltration (45, 48). Additionally, nine lncRNAs as prognostic markers for GBM patient outcome (49). Therefore, inhibition of epigenetic changes by lncRNAs has immense potential as a GBM therapy (50).

Several lncRNAs are differentially expressed in GBM relative to normal brain tissue (51). For instance, the HOX Transcript Antisense Intergenic RNA (HOTAIR) is completely undetectable in normal brain but is overexpressed in GBM tumors. HOTAIR is a lncRNA from the homeobox super family on the HOXC locus on chromosome 12q13.13 (52). It was the first transregulation lncRNA to be found and has been linked to osteoarthritis and cardiovascular disease in addition to multiple cancers (53). HOTAIR interacts with chromatin modeling complexes, which consequently leads to gene regulation and the promotion of tumor cell invasion, metastasis, and maintenance of stemness in cancer cells (54). However, in specific cancers like colorectal cancer (CRC) its exact method of action is unclear. What is known is that knockdown of HOTAIR in CRC cell lines drastically reduces CRC cell proliferation, which has also been observed in mouse GBM models as well (55, 56). Additionally, HOTAIR also plays a role in drug sensitivity in CRC cells. Knockdown of HOTAIR demonstrates that CRC cells display increased sensitivity to Cisplatin, a chemotherapy agent, and HOTAIR was observed to be upregulated in drug resistant CRC cell lines (57).

We and others determined whether HOTAIR levels were significantly correlated with GBM tumors and GBM serum (52, 54, 58). Quantitative real-time-PCR (qRT-PCR) was conducted to detect HOTAIR levels in 15 pairs of GBM tissue and GBM serum samples. The result of the study demonstrated that not only is HOTAIR dysregulated and that the dysregulated lncRNA facilitates GBM proliferation, but that there are also higher levels of HOTAIR in serum exosomes of GBM patients (52). This association between HOTAIR and GBM proliferation and expansion has been demonstrated by others as well in GBM patients *in vivo* (58). Additionally, it was discovered that HOTAIR mediates the ability of GBM cells to migrate and invade through membranes *in vitro* (56). Therefore, there is considerable evidence that not only is HOTAIR related to cancer proliferation, but that it is also an independent negative prognostic marker in GBM (54, 58).

**Figure 2:**
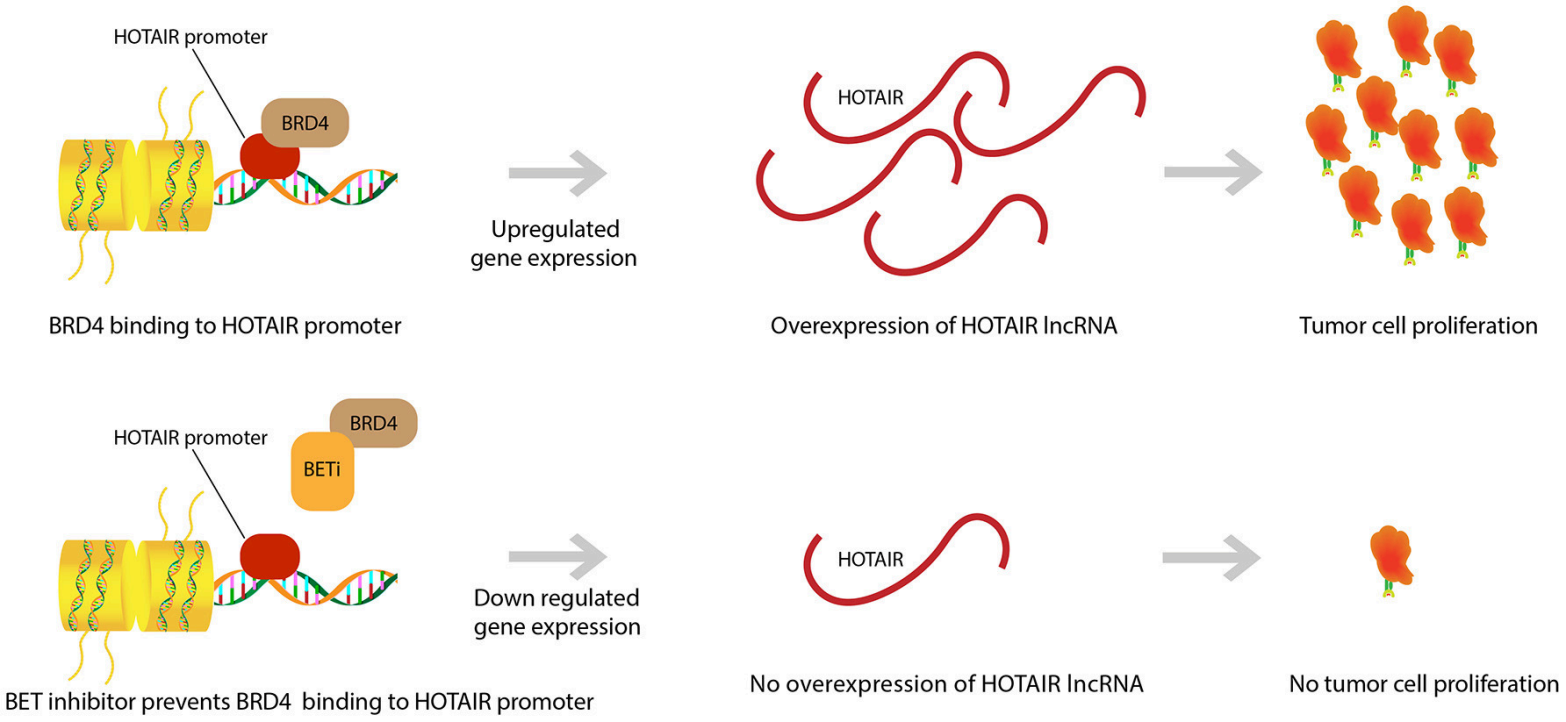
BET inhibitor drugs and HOTAIR level regulation. BRD4 binding to HOTAIR promoter region causes an upregulation in gene expression for the lncRNA HOTAIR. This overexpression in HOTAIR is associated with proliferation and expansion of GBM tumor cells. BET inhibitors prevent binding of BRD4 to the HOTAIR promoter and yield a down regulation in HOTAIR expression. Subsequently, there is prevention of tumor cell proliferation.

We demonstrated that HOTAIR is part of a proliferative pathway controlled by the bromodomain and extra-terminal domain (BET) epigenetic reader proteins (Figure 2). One such protein, Bromodomain Containing 4 (BRD4), was shown to bind to the HOTAIR promoter and in doing so, controlled HOTAIR levels. Specifically, BRD4 binding and activation led to increased levels in HOTAIR (52). As expected then, the use of a Bromodomain and Extraterminal (BET) inhibitor, I-BET151, that reduces BRD4 binding at the HOTAIR promoter was observed to cause a consequential decrease in the expression of HOTAIR in GBM cells (52, 59). Another BET inhibitor, JQ1, was observed to induce G1 cell cycle arrest and apoptosis, which led to reduction of significant GBM genes (c-MYC, hTERT, Bcl-2, Bcl-xL, and P21CiP1/WAF1) (60). JQ1 reduces tumor growth via reduction of PD-L1 expression on tumor cells leading to less T-cell death induced by the PD-L1 pathway (36). This idea of BET inhibitors promoting T-cell immune reactions against cancer cells has been further supported by research that has shown that BET inhibitors promote T-cell infiltration in mouse models. Moreover, it was discovered that epigenetic inhibitors can even rejuvenate the ability of exhausted T-cells to infiltrate tumor cells once again (61). This further exemplifies the ability of epigenetic pathways to control neoplastic activity and highlights the therapeutic potential of epigenetic pathway modulation in GBM.

Another study involving lncRNAs looked at lncRNA LINC00470, which like HOTAIR, is overexpressed in GBM when compared to normal brain tissue. Moreover, it was reported that patients with higher levels of LINC00470 had poorer prognoses in terms of survival time when compared with patients with lower levels of LINC00470 (62). Researchers in this study investigated LINC00470 and its interaction with AKT, a serine/threonine kinase related to cell proliferation, autophagy, and survival. What was discovered was that interaction between LINC00470 and AKT caused an upregulation of AKT activation thus leading to cell proliferation and GBM tumorigenesis (62). Thus, there is strong evidence for the role of lncRNA and cancer proliferation through epigenetic interactions.

Research looking into epigenetic management of cancer has shown that epigenetic inhibitors are safe and may be effective in treating certain neoplasms. For example, Vorinostat, a histone deacetylase (HDAC) inhibitor that works by preventing the action of histone deacetylases (HDACs), has been approved for treatment of Cutaneous T-cell Lymphoma and may benefit patients suffering from prostate cancer, breast cancer, and lung cancer (63). 5-Azacytidine (5-AZA), another epigenetic inhibitor that works through prevention of the enzyme DNA methyltransferase, has been shown to be effective in treating patients with myelodysplastic syndromes (64). Additionally when used in combination with chemotherapy agents, epigenetic inhibitors, such as the DNA methyltransferase inhibitor Decitabine, and chemotherapy drugs, such as DNA synthesis inhibitors Mitoxantrone Hydrochloride, Cytarabine and Etoposide, were effective in increasing overall survival in patients suffering from relapsed or refractory acute myeloid leukemia or high-risk myelodysplastic syndromes (65). Regarding epigenetic and immunotherapy combination trials, researchers are currently assessing the viability and safety of epigenetic inhibitors, like 5-AZA, with the checkpoint inhibitor, like pembrolizumab, in combinations such as 5-AZA with pembrolizumab, entinostat (HDAC inhibitor) with pembrolizumab, and vorinostat with pembrolizumab (66).

## Conclusions and future perspectives

GBM remains an incurable disease despite increased understanding of the genetic and epigenetic pathways dysregulated in these tumors. While immunotherapy trials have shown minor improvements in overall survival, the actual increase in time for the patient is still only months and treatment is highly limited to certain subtypes of GBM. Importantly, lncRNAs can be detected in patient serum and can be used as biomarkers for efficacy of drugs in patients. Thus, it seems that the pursuit of other treatment options such as combining epigenetic pathway inhibitors along with immunotherapy treatment could bring medicine closer to treating GBM. Though it may be difficult to create a blanket treatment for all types of GBM, it is worth dedicating resources into future research of individualized patient treatment.

## Author contributions

All authors listed have made a substantial, direct and intellectual contribution to the work, and approved it for publication.

### Conflict of interest statement

The authors declare that the research was conducted in the absence of any commercial or financial relationships that could be construed as a potential conflict of interest.
